# 2558 Current efforts to increase adolescent human papillomavirus vaccination rates using school-based setting in Starr County, Texas

**DOI:** 10.1017/cts.2018.246

**Published:** 2018-11-21

**Authors:** Ana Marina Rodriguez, Kathleen Schmeler, Sapna Kaul, Yong-Fang Kuo

**Affiliations:** 1 Univeristy of Texas Medical Branch; 2 Anderson Cancer Center; 3 University of Texas Medical Branch at Galveston

## Abstract

OBJECTIVES/SPECIFIC AIMS: Improving human papillomavirus (HPV) vaccination rates ultimately decreases the morbidity and mortality of HPV-associated diseases. A school-based program was piloted in the Rio Grande City Consolidated Independent School District (RGCCISD) to increase HPV vaccination. METHODS/STUDY POPULATION: We assessed baseline HPV vaccination; surveyed 622 parents of eligible children aged ≥9 years; and piloted and developed a school-based HPV education and vaccination program in 1 middle school in 2017 and 4 additional middle schools in 2018. The parent survey included (1) demographic information, (2) an assessment of parental knowledge about the HPV vaccine, and (3) information about their children and HPV vaccine experience. Results of the parent survey and pilot program are in progress. RESULTS/ANTICIPATED RESULTS: As of 9/1/2016, 20.4% of the 7527 RGCCISD eligible students (≥9 years) had completed the HPV vaccine. Baseline completion rates were higher for RGCCISD students aged 12–14 years compared with students aged 9–11 and ≥15 years (28.4% vs. 16.5%). Baseline completion rates for RGCCISD adolescents were substantially lower than those reported in NIS-Teen and for Texas (42% females and 28% males for NIS-Teen vs. 41% and 24% for the state of Texas). DISCUSSION/SIGNIFICANCE OF IMPACT: Initial results show that engagement with key stakeholders is important and schools are a great venue for delivering and increasing HPV vaccination.

